# Does digital device software lead to exclusion? Investigating a portable metabolic analysis system and the input of sex data on physiological parameters

**DOI:** 10.3389/fdgth.2025.1541083

**Published:** 2025-06-06

**Authors:** James W. Navalta, Olivia R. Perez, Michael W. H. Wong, Dustin W. Davis

**Affiliations:** Exercise Physiology Laboratory, Department of Kinesiology and Nutrition Sciences, University of Nevada, Las Vegas, Las Vegas, NV, United States

**Keywords:** digital health, metabolic analysis, gender inclusion, wearable technology, exercise physiology

## Abstract

**Background:**

Digital health devices have enhanced healthcare accessibility, but their design may unintentionally exclude gender diverse people. This study examines whether the input of binary sex data in a portable metabolic analysis system (COSMED K5) impacts the accuracy of physiological measurements during self-paced exercise.

**Methods:**

Twenty adult participants (10 females, 10 males) completed two identical self-paced walking and running protocols with sex data alternately input as female or male in the device software. Key metabolic and pulmonary variables, including VO_2_, VCO_2_, ventilation, respiratory exchange ratio (RER), respiratory rate, and energy expenditure, were measured. Statistical comparisons evaluated differences between conditions.

**Results:**

No differences were observed in any measured variables between the female and male conditions during walking or running (*p* > 0.05). Correlations between conditions were strong (*r* = 0.73–0.98).

**Conclusion:**

The COSMED K5 device does not utilize binary sex input to alter physiological outputs, confirming that these data remain unaffected by this demographic variable. However, the limitation of binary sex options in the device software represents a barrier to inclusivity for gender diverse people. Device manufacturers are encouraged to update software with more inclusive options, aligning with recommendations for equitable research practices and addressing existing knowledge gaps in sport and exercise science.

## Introduction

The incorporation of digital devices and wearables for personal health data has the potential to reduce barriers to health access (1). Perceived barriers resulting in delayed care include discrimination and access (i.e., ability to make appointments, transportation to care location, limited care hours) (2). Digital devices, as inanimate objects, do not discriminate while increasing patient engagement by facilitating the sharing of data with providers (3). Additionally, device sensors can be connected to care providers around the clock to provide real-time personalized medicine in a remote format (4), eliminating the need to make appointments or travel to care locations. While digital devices hold promise, we acknowledge that cost (5) and greater adoption remain barriers to full integration into health care (6).

An important component of health is cardiorespiratory fitness. Cardiorespiratory fitness reflects the ability of the circulatory and respiratory systems to provide skeletal muscle with oxygen necessary for energy production during exercise and physical activity (7). Determining cardiovascular fitness is important because moderate to high levels are associated with a reduced risk of all-cause mortality (8), regardless of adiposity, age, alcohol intake, ethnicity, and smoking status (9). Cardiorespiratory fitness is traditionally measured by laboratory-based metabolic analysis systems and the collection of expired gasses during incrementally increasing intensity exercise tests (10). Advances in technology have resulted in the development of valid wearable portable metabolic analysis systems (11) which allows sport and exercise scientists to obtain measurements outside the laboratory.

Additional demographic variables commonly reported in the sport and exercise science literature include sex and gender. Sex refers to biological factors (i.e., genetic, hormonal, and or anatomical traits) (12) while gender is a social construct with roles and activities that are learned through a socialization process (13). For this reason, it is recommended that data on sex and gender be collected in a two-step process, (1) sex assigned at birth, and (2) current gender (14). Acknowledging diversity in gender is important, and includes people who are non-binary, transgender, and gender fluid across many indigenous cultures (i.e., Two Spirit in North America, Hijra in India, fa'afafine in Samoa) (15). There is a need for greater inclusion in sport and exercise science research, particularly when considering sex and gender diverse people (16). A study of over 800 published papers on exercise science-related topics reported that three investigations collected participant sex or gender with options other than the female-male binary (17). Allowing participants to identify their sex and gender in inclusive terms is important because misgendering (when an individual is described using terminology that is inconsistent with their gender identification) can affect statistical outcomes and interpretation of data in typical sample sizes employed in sport and exercise science research (18). It is possible that a barrier to inclusion for sport and exercise scientists exists in the software and equipment most regularly used.

Metabolic analysis systems are a common piece of equipment in exercise physiology laboratories and provide data for many exercise investigations (19). The software connected to these devices require the input of sex data, and to our knowledge, is limited to the binary options of female or male. Because metabolic analysis systems and the derived data should not be affected by the sex selected in the software, it was hypothesized that there would be no differences in metabolic or pulmonary data between identical exercise bouts conducted by the same individual when categorized as a female or male. Thus, the purpose of this investigation was to test the hypothesis by having the same participants perform identical self-paced walking bouts and running bouts under the female and male categories in the software.

## Materials and methods

### Participants

An effect size (*r* = 0.9397) was calculated using reported differences in energy expenditure between women and men during exercise (20). An *a priori* power analysis was conducted in G*Power (21) using correlation: bivariate normal model statistical test (exact test family), an α error of probability of 0.05, and a power (1−β error of probability) of 0.95 indicating a total sample size of seven participants. To be conservative, we recruited and tested the number of participants most commonly used in sport and exercise science (*N* = 20) (17). The investigation was approved by the institution's Institutional Review Board (#2023-525) and carried out fully in accordance to the ethical standards of the *International Journal of Exercise Science* (22).

Twenty adult participants (self-identified sex: female *n* = 10, male *n* = 10, identified otherwise *n* = 0) took part in this study after submitting an online informed consent form via Qualtrics (Provo, UT), including a health risk questionnaire to determine eligibility for this study. Participants were recruited from the University of Nevada, Las Vegas campus and surrounding communities. Participant demographic information (arithmetic mean ± standard deviation) included age (years) 24.5 ± 7.5, height (cm) 168.3 ± 9.2, and body mass (kg) 68.8 ± 13.7.

### Protocol

A randomization chart (Google Sheets, Mountainview, CA) was generated and utilized to determine the order in which sex was input into the Omnia software (Rome, Italy) demographics profile and COSMED K5 portable metabolic analysis system (Rome, Italy). The randomization chart determined whether a participant sex was entered into the Omnia software as “female” during the initial trial, or whether the participant sex was entered as “male” (all other demographic information was consistent between trials). A Polar H10 heart rate monitor (Polar Electro Inc., Kempele, Finland) was paired with the COSMED K5 to collect heart rate data in conjunction with metabolic and pulmonary variables. The metabolic variables collected included relative VO_2_ (ml·kg^−1^·min^−1^), absolute VO_2_ (L·min^−1^), VCO_2_ (L·min^−1^), ventilation [VE (L·min^−1^)], respiratory exchange ratio (RER), respiratory rate in breaths per minute [RR (BPM)], and accumulated total kilocalories (kcal).

The study was completed on a single testing day. Participant resting heart rate was obtained, followed by facemask fitting for the COSMED K5. Resting heart rate was determined by having participants sit quietly in a comfortable position for a minimum of five minutes, during which heart rate was continuously monitored until it was stabilized with no interruptions. The lowest observed heart rate during this period was recorded as the resting heart rate. A facemask was chosen for participant comfort and because no difference in metabolic data have been reported when compared to a traditional mouthpiece apparatus (23). Participants selected preferred walking and running speeds on a treadmill (WOODWAY 4Front, Waukesha, WI) through a blinded procedure over three trials each (24). During each trial, participants increased the treadmill speed from 0 m·min^−1^ to their preferred walking/running speed, which they perceived they could maintain for five minutes. Once preferred speeds from the three trials were collected, the respective values were averaged to determine the selected walking (mean = 64.9 ± 18.2 m·min^−1^) and running speeds (mean = 130.2 ± 29.0 m·min^−1^) that were utilized for the remainder of the study (24). Each participant performed only a single walking speed during the walking trials and a single running speed during the running trials.

**Figure 1:**
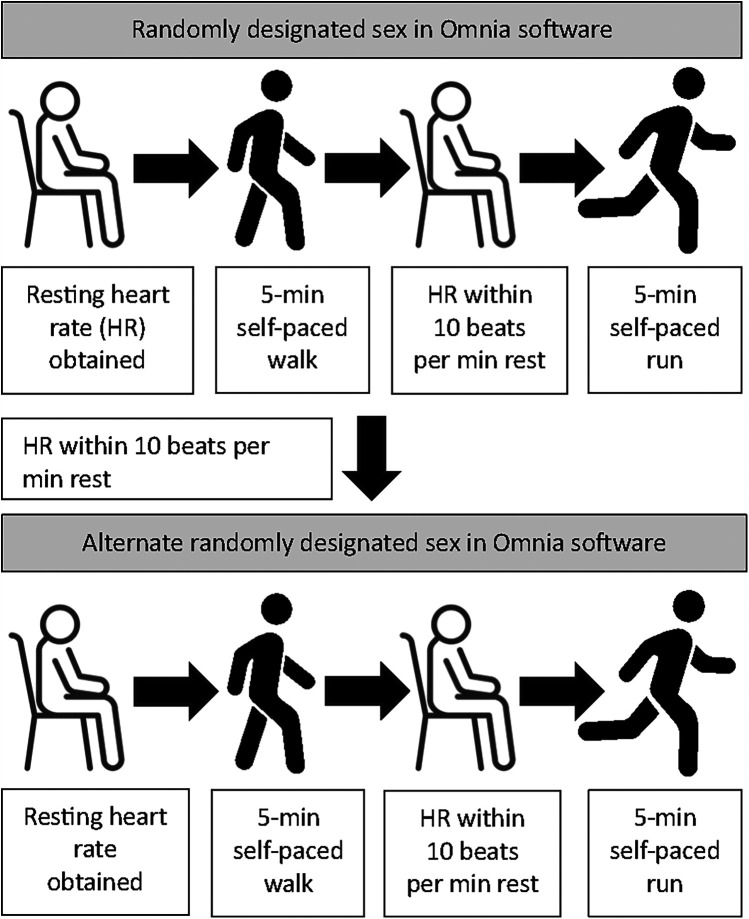
Study protocol depicting rest, self-paced walking, and self-paced running under randomly designated sex available in the omnia software environment. HR, heart rate. Artist attribution: Nigar Novruzova (vecteezy.com). Icons reproduced from: “Sit icon” by cube29; “Vector illustration of man stands, walk and run icon set” by Nigar Novruzova, licensed under Free License.

The facemask was attached to the participant and, after checking for air leakage, the COSMED K5 was securely placed in front of the participant on the treadmill. Participants completed a total of 20 minutes of exercise, divided into two five-minute walking and two five-minute running bouts (see Figure 1). Each participant completed bouts for walking and running under the female and male sex in the available Omnia software associated with the COSMED K5. Between each bout, participants were guided off the treadmill and instructed to sit for a rest period until their heart rate was within 10 beats per minute of their resting heart rate. The mean rest time between the walk and the run was 3.6 ± 3.9 minutes. When the first walking and running trials under the randomly designated sex were completed, a new demographics profile was created for the alternate binary sex option in the software using the same age, height, and body mass, and the protocol was repeated. During this time, participants were instructed to sit and rest until the heart rate was within 10 beats per minute of the resting heart rate (mean time = 6.3 ± 3.5 minutes).

### Statistical analysis

Statistical tests (IBM SPSS Statistics, Version 29.0.2.0, Armonk, NY) were conducted to assess potential differences in metabolic and pulmonary variables between input for the female sex condition and the male sex condition, specifically a dependent *t*-test for each dependent variable. Data were checked for normality using the Shapiro–Wilk test. *P*-values < 0.05 were considered significant. Effect sizes were calculated through Cohen's *d*, with negligible = 0.0–0.2, small = 0.2–0.49, medium = 0.5–0.79, and large ≥0.8 (25). Correlations between the female and male conditions were evaluated using Pearson product-moment correlation coefficients (*r*) for each dependent variable and *r*^2^ as a measure of effect size.

## Results

All data met the assumptions for normality. No differences between the female and male conditions were observed for any metabolic or pulmonary variables during self-paced walking (see Table 1). The shared variance was between 54% and 96% (see Table 1).

**Table 1:** Metabolic and pulmonary variables during self-paced walking when sex was input as female in the omnia software environment and when the sex was input as male.

Variable	Female condition	Male condition	*p*-value	Cohen's *d*	Pearson's *r*	*r* ^2^
VO_2_ (ml·kg^−1^·min^−1^)	13.53 (3.04)	13.76 (3.35)	0.26	0.26	0.9658	0.9328
VO_2_ (L·min^−1^)	0.92 (0.22)	0.93 (0.21)	0.44	0.18	0.9724	0.9455
VCO_2_ (L·min^−1^)	0.72 (0.20)	0.73 (0.20)	0.08	0.42	0.9801	0.9607
VE (L·min^−1^)	23.37 (5.62)	24.30 (4.75)	0.06	0.47	0.9404	0.8843
RER	0.77 (0.06)	0.79 (0.64)	0.06	0.45	0.9234	0.8527
RR (bpm)	21.37 (5.16)	22.78 (3.81)	0.09	0.39	0.7344	0.5394
EE (kcal)	21.95 (5.18)	22.6 (5.61)	0.09	0.40	0.9575	0.9168

VO_2_, oxygen uptake; VCO_2_, carbon dioxide production; VE, pulmonary ventilation; RER, respiratory exchange ratio; RR, respiratory rate; bpm, breaths per minute; EE, energy expenditure; kcal, kilocalories.

Similar to walking, no differences between the female and male conditions were observed for any metabolic or pulmonary variables during self-paced running (see Table 2). The shared variance was between 67% and 97% (see Table 2).

**Table 2:** Metabolic and pulmonary variables during self-paced running when sex was input as female in the omnia software environment and when the sex was input as male.

Variable	Female condition	Male condition	*p*-value	Cohen's *d*	Pearson's *r*	*r* ^2^
VO_2_ (ml·kg^−1^·min^−1^)	33.19 (6.62)	33.08 (6.49)	0.76	0.07	0.9728	0.9463
VO_2_ (L·min^−1^)	2.25 (0.54)	2.24 (0.49)	0.53	0.14	0.9852	0.9707
VCO_2_ (L·min^−1^)	1.97 (0.53)	1.95 (0.52)	0.46	0.17	0.9489	0.9004
VE (L·min^−1^)	59.52 (17.44)	60.04 (14.82)	0.64	0.11	0.9653	0.9319
RER	0.87 (0.06)	0.88 (0.05)	0.21	0.29	0.8395	0.7047
RR (bpm)	34.38 (8.71)	34.54 (8.17)	0.90	0.03	0.8165	0.6666
EE (kcal)	52.30 (12.46)	52.05 (10.48)	0.71	0.09	0.9828	0.9659

VO_2_, oxygen uptake; VCO_2_, carbon dioxide production; VE, pulmonary ventilation; RER, respiratory exchange ratio; RR, respiratory rate; bpm, breaths per minute; EE, energy expenditure; kcal, kilocalories.

According to the randomization schema, 14 participants (*n* = 7 female, *n* = 7 male) began the study under the condition that aligned with their self-identified sex (i.e., female participant randomized into the female condition first), while 6 participants (*n* = 3 female, *n* = 3 male) began the study under the condition that was not aligned with their self-identified sex (i.e., female participant randomized into the male condition first). No differences for any variable were observed when the aligned with sex condition was compared to the condition not aligned with participants' sex for walking (see Table 3) or running (see Table 4).

**Table 3:** Metabolic and pulmonary variables during self-paced walking when condition aligned with participant’ sex and when condition did not align with participants’ sex.

Variable	Sex aligned	Sex not aligned	*p*-value	Cohen's *d*	Pearson's *r*	*r* ^2^
VO2 (ml·kg^−1^·min^−1^)	13.60 (2.97)	13.70 (3.41)	0.63	0.11	0.9684	0.9378
VO2 (L·min^−1^)	0.92 (0.21)	0.92 (0.21)	0.87	0.04	0.9701	0.9411
VCO2 (L·min^−1^)	0.72 (0.20)	0.73 (0.20)	0.55	0.14	0.9766	0.9537
VE (L·min^−1^)	23.49 (5.26)	24.17 (5.16)	0.16	0.33	0.9202	0.8468
RER	0.78 (0.07)	0.78 (0.06)	0.32	0.23	0.9213	0.8488
RR (bpm)	21.62 (4.93)	22.53 (4.18)	0.28	0.25	0.6865	0.4712
EE (kcal)	22.20 (5.51)	22.35 (5.30)	0.71	0.09	0.9480	0.8988

VO_2_, oxygen uptake; VCO_2_, carbon dioxide production; VE, pulmonary ventilation; RER, respiratory exchange ratio; RR, respiratory rate; bpm, breaths per minute; EE, energy expenditure; kcal, kilocalories.

**Table 4:** Metabolic and pulmonary variables during self-paced running when condition aligned with participants’ sex and when condition did not align with participants’ sex.

Variable	Sex aligned	Sex not aligned	*p*-value	Cohen's *d*	Pearson's *r*	*r* ^2^
VO2 (ml·kg^−1^·min^−1^)	33.03 (6.71)	33.25 (6.41)	0.52	0.15	0.9741	0.9488
VO2 (L·min^−1^)	2.24 (0.51)	2.26 (0.52)	0.42	0.18	0.9819	0.9641
VCO2 (L·min^−1^)	1.94 (0.54)	1.98 (0.52)	0.24	0.27	0.9518	0.9059
VE (L·min^−1^)	59.47 (15.10)	60.08 (17.20)	0.59	0.12	0.9609	0.9234
RER	0.88 (0.05)	0.88 (0.06)	0.95	0.02	0.8243	0.6795
RR (bpm)	34.08 (8.00)	34.84 (8.84)	0.51	0.15	0.8226	0.6767
EE (kcal)	51.75 (10.85)	52.60 (12.13)	0.19	0.31	0.9769	0.9544

VO_2_, oxygen uptake; VCO_2_, carbon dioxide production; VE, pulmonary ventilation; RER, respiratory exchange ratio; RR, respiratory rate; bpm, breaths per minute; EE, energy expenditure; kcal, kilocalories.

The present study did not aim to evaluate sex differences, so we did not test for them. However, to align with the Sex and Gender Equity in Research (SAGER) guidelines (26) and support future meta-analyses, we present disaggregated metabolic and pulmonary data in Table 5. Table 6 presents disaggregated metabolic and pulmonary data when participants’ self-identified sex aligned with the randomized condition, and when it did not align.

**Table 5:** Disaggregated metabolic and pulmonary data of females (*n* = 10) walking and running in the female and male condition, and males (*n* = 10) in walking and running in the female and male condition.

Variable	Females walking		Females running	
	Female condition	Male condition	Female condition	Male condition
VO_2_ (ml·kg^−1^·min^−1^)	13.44 (3.61)	13.77 (4.25)	31.44 (7.30)	31.56 (6.91)
VO_2_ (L·min^−1^)	0.83 (0.16)	0.84 (0.16)	1.94 (0.40)	1.95 (0.35)
VCO_2_ (L·min^−1^)	0.62 (0.14)	0.65 (0.15)	1.65 (0.31)	1.67 (0.26)
VE (L·min^−1^)	20.78 (4.53)	22.38 (4.23)	51.05 (10.02)	52.18 (10.15)
RER	0.75 (0.04)	0.77 (0.04)	0.85 (0.04)	0.86 (0.05)
RR (bpm)	21.71 (6.12)	24.03 (3.64)	33.71 (10.32)	34.63 (10.61)
EE (kcal)	19.80 (4.34)	20.60 (5.02)	44.70 (7.65)	45.30 (7.15)
Variable	Males walking		Males running	
	Female condition	Male condition	Female condition	Male condition
VO_2_ (ml·kg^−1^·min^−1^)	13.62 (2.55)	13.75 (2.36)	34.94 (5.70)	34.61 (6.00)
VO_2_ (L·min^−1^)	1.01 (0.24)	1.02 (0.22)	2.56 (0.48)	2.53 (0.44)
VCO_2_ (L·min^−1^)	0.81 (0.21)	0.82 (0.21)	2.29 (0.53)	2.22 (0.58)
VE (L·min^−1^)	25.97 (5.59)	26.21 (4.65)	67.98 (19.56)	67.90 (14.95)
RER	0.80 (0.07)	0.81 (0.08)	0.89 (0.07)	0.90 (0.05)
RR (bpm)	21.03 (4.31)	21.53 (3.73)	35.05 (7.25)	34.44 (5.33)
EE (kcal)	24.10 (5.24)	24.60 (5.70)	59.90 (11.87)	58.80 (8.92)

VO_2_, oxygen uptake; VCO_2_, carbon dioxide production; VE, pulmonary ventilation; RER, respiratory exchange ratio; RR, respiratory rate; bpm, breaths per minute; EE, energy expenditure; kcal, kilocalories.

**Table 6:** Disaggregated metabolic and pulmonary data walking and running in the condition where sex was aligned, and in the condition where sex was not aligned.

Variable	Walking—sex aligned		Running—sex aligned	
	Female (*n* = 10)	Male (*n* = 10)	Female (*n* = 10)	Male (*n* = 10)
VO_2_ (ml·kg^−1^·min^−1^)	13.44 (3.61)	13.75 (2.36)	31.44 (7.30)	34.61 (6.01)
VO_2_ (L·min^−1^)	0.83 (0.16)	1.02 (0.22)	1.94 (0.40)	2.53 (0.45)
VCO_2_ (L·min^−1^)	0.62 (0.14)	0.82 (0.21)	1.65 (0.31)	2.22 (0.58)
VE (L·min^−1^)	20.78 (4.53)	26.21 (4.65)	51.05 (10.02)	67.90 (14.95
RER	0.75 (0.04)	0.81 (0.08)	0.85 (0.04)	0.90 (0.05)
RR (bpm)	21.71 (6.12)	21.53 (3.73)	33.71 (10.32)	34.44 (5.34)
EE (kcal)	19.80 (4.34)	24.60 (5.70)	44.70 (7.65)	58.80 (8.92)
Variable	Walking—sex not aligned		Running—sex not aligned	
	Female (*n* = 10)	Male (*n* = 10)	Female (*n* = 10)	Male (*n* = 10)
VO_2_ (ml·kg^−1^·min^−1^)	13.77 (4.25)	13.62 (2.55)	31.56 (6.91)	34.94 (5.70)
VO_2_ (L·min^−1^)	0.84 (0.16)	1.01 (0.24)	1.95 (0.35)	2.56 (0.48)
VCO_2_ (L·min^−1^)	0.65 (0.15)	0.81 (0.21)	1.67 (0.26)	2.29 (0.53)
VE (L·min^−1^)	22.38 (4.23)	25.97 (5.59)	52.18 (10.15)	67.98 (19.56)
RER	0.77 (0.04)	0.80 (0.07)	0.86 (0.05)	0.89 (0.07)
RR (bpm)	24.03 (3.64)	21.03 (4.31)	34.63 (10.61)	35.05 (7.25)
EE (kcal)	20.60 (5.01)	24.10 (5.24)	45.30 (7.15)	59.90 (11.87)

VO_2_, oxygen uptake; VCO_2_, carbon dioxide production; VE, pulmonary ventilation; RER, respiratory exchange ratio; RR, respiratory rate; bpm, breaths per minute; EE, energy expenditure; kcal, kilocalories.

## Discussion

We hypothesized that a portable metabolic analysis system measuring metabolic and pulmonary variables would not be affected by participant sex entered into the software environment, and as such, no differences in the variables would be observed between the female or male sex categories available in the software during self-paced walking or self-paced running. Our hypothesis was supported, as oxygen uptake (expressed in absolute and relative terms) carbon dioxide output, respiratory exchange ratio, pulmonary ventilation, respiratory rate, and accumulated kilocalories were unaffected by the sex data entered into the device software.

These results provide evidence that, while differences among females and males may be present for metabolic and pulmonary variables during exercise (20, 27), the COSMED K5 and accompanying Omnia software designed to measure such variables do not utilize the input of demographic sex data to measure the metabolic or pulmonary variables investigated in the present study. We believe that any deviation between conditions is due to the usual physiological variation that occurs between bouts of exercise (28, 29). However, it must be pointed out in the present study that workload in terms of walking and running speed was maintained from one bout to the next. Because the device software limits the sex input to two options (female and male), a potential consequence is an unintentional barrier, engineered into the software, to the inclusion of sex and gender diverse people. We suggest a similar unintentional barrier may exist with much of the equipment found in exercise physiology (i.e., electrocardiogram machines, electromyography systems, blood lactate analyzers, spirometers) and biomechanics (motion capture systems, force platforms, isokinetic dynamometers) laboratories and the accompanying software running these devices. While testing similar to what was conducted in the current investigation is likely unnecessary for all equipment, an opportunity exists, particularly with respect to sex and gender input options, for device software to be updated to be more inclusive of people who are sex and gender diverse. These findings may have regulatory implications, as they suggest that a more critical evaluation of sex-based algorithms in medical devices and digital health tools is warranted. Future guidelines should prioritize evidence-based inclusion of demographic variables.

Belief in sex differences in energy expenditure and resting metabolic rate is well-entrenched dogma (30). The Harris and Benedict equation for estimating basal metabolic rate was published in 1918 and assumed a difference between females and males of up to 7% (31). The Mifflin-St Joer equation for estimating resting metabolic rate was published in 1990 and assumed a sex difference of 166 kcal (32). With respect to physical activity, sex differences in energy expenditure have been reported in individuals completing a three-month wilderness expedition; however, it should be noted that measures were estimated from actigraphy (33). Additionally, males have been reported to expend significantly greater energy expenditure than females while playing golf, however kcal was derived as estimates via heart rate devices rather than indirect calorimetry (34). The energy estimations for physical activity and exercise derived from actigraphy and wearable device estimations are likely influenced by the same assumptions embedded within most resting metabolic rate equations. While it is outside the scope of this investigation to explore underlying assumptions, we can state that the portable metabolic analysis system utilized in the present study was not influenced by the same limitation, as no differences were observed between sex input as an independent variable. Wearable and other digital device manufacturers whose devices measure or estimate energy expenditure may wish to reconsider certain assumptions and make their software more inclusive. Additionally, many fitness wearables require users to input their sex to create a profile, but if the measured outcome is step count, our research suggests the input is not needed.

A knowledge gap in the sport and exercise science literature among females and males has been documented because males make up the majority of participants tested, at around 65% (17, 35, 36). The consequence of this knowledge gap is a lack of appropriate scientific recommendations for females in areas such as training, recovery, and rehabilitation (36). We propose that a much wider chasm exists for sex and gender diverse people, such as people who are intersex, transgender, and/or non-binary. Because of this, a leading professional organization in the field, the American College of Sports Medicine, is only able to provide a single paragraph in recommendation to gender diverse people among the over 500-page text (37). While there is a disparity of reporting sex and gender diverse people in the sport and exercise science literature (17), it is possible that a true lack of inclusion may not be present. Similar to what we report in the present study, a barrier may be present at the software- and equipment-level running through the study design, which results in data on sex and gender diverse people not being collected or ultimately reported in the literature. We have suggested that investigators consider how sex and gender data are collected (18). If researchers rely on obtaining these data from their connected devices and software, having insufficient options for sex and gender maintains the present knowledge gap and exclusivity of the sport and exercise science literature.

This study is not without limitations. The authors acknowledge that while we propose an opportunity for greater inclusivity in sport and exercise science research exists, the current study only tested participants who identified as cisgender females or males. While we did not exclude gender diverse individuals from participating, our findings cannot directly speak to the experiences of those outside the binary framework. A potential limitation may be in our recruitment and enrollment methodology, which could have reduced the likelihood of participation by people who identify as sex or gender diverse. To address this, future studies could employ more targeted recruitment of gender diverse individuals by partnering with LGBTQ + community groups or gender clinics.

In conclusion, we report that an individual's sex, as designated in the Omnia software and associated COSMED K5 portable metabolic analysis system, does not affect measures of relative and absolute oxygen uptake, carbon dioxide production, ventilation, respiratory exchange ratio, respiratory rate, or energy expenditure. From a practical standpoint, researchers who inadvertently enter a participants sex incorrectly into the Omnia software can be reassured the mistake will have no adverse effects on the outcome variables. We propose that limiting the input of sex options to the female-male binary is a barrier to researchers’ ability to include sex and gender diverse people. Manufacturers of digital devices and equipment whose measurements or estimations are not affected by sex could remove this barrier by updating software with inclusive options, potentially enabling the reporting of disaggregated data as recommended by the SAGER guidelines (26). These actions could help to address the knowledge gap present for people who are sex and gender diverse in sport and exercise science research. Until digital devices allow for more inclusive sex and gender options, researchers are encouraged to be intentional in their approach for collecting sex and gender using a two-step process [sex, acknowledging more than binary options (i.e., intersex); and gender, allowing for an open-response option].

## Data Availability

The raw data supporting the conclusions of this article will be made available by the authors, without undue reservation.
